# Homogeneity in immune features between colorectal liver metastases better identifies patients with good prognosis compared to pathological response to preoperative chemotherapy

**DOI:** 10.1080/2162402X.2023.2253642

**Published:** 2023-09-14

**Authors:** David Henault, David Stephen, Pierre-Antoine St-Hilaire, Nouredin Messaoudi, Franck Vandenbroucke-Menu, Eve Simoneau, Zhixia Rong, Marylène Plasse, Richard Létourneau, André Roy, Michel Dagenais, Réal Lapointe, Bich Nguyen, Anne-Marie Mes-Masson, G. Soucy, Simon Turcotte

**Affiliations:** a Hepato-Pancreato-Biliary Surgery Service, Centre hospitalier de l’Université de Montréal, Montréal, Québec, Canada; b Cancer Axis, Centre de recherche du Centre Hospitalier de l’Université de Montréal/Institut du cancer de Montréal, Montréal, Québec, Canada; c Department of Pathology, Centre hospitalier de l’Université de Montréal, Montréal, Québec, Canada; d Department of Surgery, Vrije Universiteit Brussel (VUB), Universitair Ziekenhuis Brussel (UZ Brussel) and Europe Hospitals, Brussels, Belgium; e Department of Medicine, Université de Montréal, Montreal, QC, Canada

**Keywords:** Colorectal liver metastasis, immune score, prognostic biomarker, tumor heterogeneity, tumor infiltrating lymphocytes

## Abstract

In colorectal cancer liver metastases (CRLM), the density of tumor-infiltrating lymphocytes, the expression of class I major histocompatibility complex (MHC-I), and the pathological response to preoperative chemotherapy have been associated with oncological outcomes after complete resection. However, the prognostic significance of the heterogeneity of these features in patients with multiple CRLMs remains under investigation. We used a tissue microarray of 220 mismatch repair-gene proficient CRLMs resected in 97 patients followed prospectively to quantify CD3^+^ T cells and MHC-I by immunohistochemistry. Histopathological response to preoperative chemotherapy was assessed using standard scoring systems. We tested associations between clinical, immunological, and pathological features with oncologic outcomes. Overall, 29 patients (30.2%) had CRLMs homogeneous for CD3+ T cell infiltration and MHC-I. Patients with immune homogeneous compared to heterogeneous CRLMs had longer median time to recurrence (TTR) (30 vs. 12 months, *p* = .0018) and disease-specific survival (DSS) (not reached vs. 48 months, *p* = .0009). At 6 years, 80% of the patients with immune homogeneous CRLMs were still alive. Homogeneity of response to preoperative chemotherapy was seen in 60 (61.9%) and 69 (80.2%) patients according to different grading systems and was not associated with TTR or DSS. CD3 and MHC-I heterogeneity was independent of response to pre-operative chemotherapy and of other clinicopathological variables for their association with oncological outcomes. In patients with multiple CRLMs resected with curative intent, similar adaptive immune features seen across metastases could be more informative than pathological response to pre-operative chemotherapy in predicting oncological outcomes.

## Introduction

The TNM staging system is a valid tool to guide treatment decisions in non-metastatic colorectal cancer (CRC) based on expected prognosis but can be further enhanced by the Immunoscore, a compound score of tumor-infiltrating lymphocytes counted at the invasive margin (IM) and the intratumoral (IT) areas.^1^ Colorectal cancer most commonly progresses with metastases to the liver, often multiple and biologically heterogeneous, i.e. clonally distinct at the genomic^2^ and immunologic^3^ levels. Intra-patient metastatic heterogeneity complexifies outcome predictions and may be the main limitation to the success of precision medicine as well as immune checkpoint blockade therapy, as shown in metastatic melanoma for example.^4^

Patients with colorectal cancer liver metastases (CRLMs) may be amenable to curative intent partial hepatectomy. Combined with perioperative systemic chemotherapy, this approach can cure approximately 20% of the patients, but 80% recur with 2 years of hepatectomy.^5^ The Clinical Risk Score (CRS),^6^ the oncogenic KRAS status,^7^ and the pathological response to pre-operative chemotherapy^8,9^ can help identify patients at higher risk of recurrence, but are not currently used to guide adjuvant treatment or surveillance. Importantly, immune cell infiltration at the IM and IT in resected CRLMs was reported as a better predictor of time to recurrence and survival compared to these clinical, genomic, and pathological features.^10^ In the underlying studies, in case of multiple CRLMs, the metastasis least infiltrated by immune cells was the best to consider in categorizing patients prognostically.^11,12^ We also found that the presence of even a single CRLM poorly infiltrated by T cells in the context of preserved antigen presentation potential, inferred by Major histocompatibility Complex class I (MHC-I) expression was closely associated with poor outcome.^13^ These findings support the concept that the “worst metastasis,” by escaping immune surveillance or immunogenic cell death, may drive poor outcomes, but did not address whether intra-patient inter-metastatic immune heterogeneity was in itself associated with outcomes, compared to heterogeneity in pathological response to chemotherapy.

The goal of this study was to describe inter-tumoral immune heterogeneity in patients with multiple CRLMs, assessed by CD3 and MHC-I quantification and to evaluate its prognostic significance.

## Methods

### Cohort

Between January 2011 and 2014, 214 patients with synchronous, metachronous or recurrent, mismatch-repair gene proficient CRLMs (*n* = 391) treated with curative intent partial hepatectomy consented to contribute to an institutional biobank with clinicopathological annotations and prospective follow-up (updated to 10/2017), approved by the local institutional review board (No. 09.237). Patients (*n* = 97) with more than one CRLM (*n* = 220) at their index hepatectomy were selected to create the current study cohort. The standard peri-operative systemic treatment consisted of 5FU, leucovorin combined with oxaliplatin or irinotecan-based chemotherapy, with or without anti-VEGF therapy, administered before and/or after surgery, totaling 12 cycles on average.^14^ Pathological response to chemotherapy was scored according to the Rubbia-Brandt Tumor Regression Grade (TRG)^8^ and the Blazer score^9^ by a trained pathologist for each studied metastasis on whole slides.^8^ Relevant variables were included, such as demographic and clinical data, number and size of CRLM, recurrence timing and pattern, survival, treatment, and type of primary tumor.

### Immunohistochemistry

Individual pathology formalin-fixed paraffin-embedded (FFPE) blocs were reviewed by a trained pathologist to identify representative sections containing both CRLM and normal adjacent liver. Tissue microarrays (TMA) were constructed by sampling selected tissue blocs using 0.6 mm diameter biopsies in sixplicate for both the invasive margin (IM) and the intratumoral (IT) areas. The TMA was built with one metastasis for patients with unique CRLM, the two largest metastases for those with two to three CRLMs, and the three largest metastases for those with more than three CRLMs. TMA blocks were cut to 3 μm sections, deparaffinized, rehydrated in graded alcohol and stained with biotinylated secondary antibodies. Staining was done for CD3 (F7.2.38, Dako, Carpinteria, CA) and MHC-I (HC10, mouse monoclonal anti‐human HLA class I heavy chain, provided by H. Ploegh, Whitehead Institute, Cambridge, MA; 1:1,000; 1 h). The MHC-I antibody is a validated mouse anti-human monoclonal antibody that binds to MHC-I heavy chains, preferentially for the HLA-B and HLA-C molecules, and seven HLA-As. Hematoxylin was used to stain nuclei, and recurrent eosin staining was used for normal liver, lymph nodes, and tonsils.

**Figure 1.**
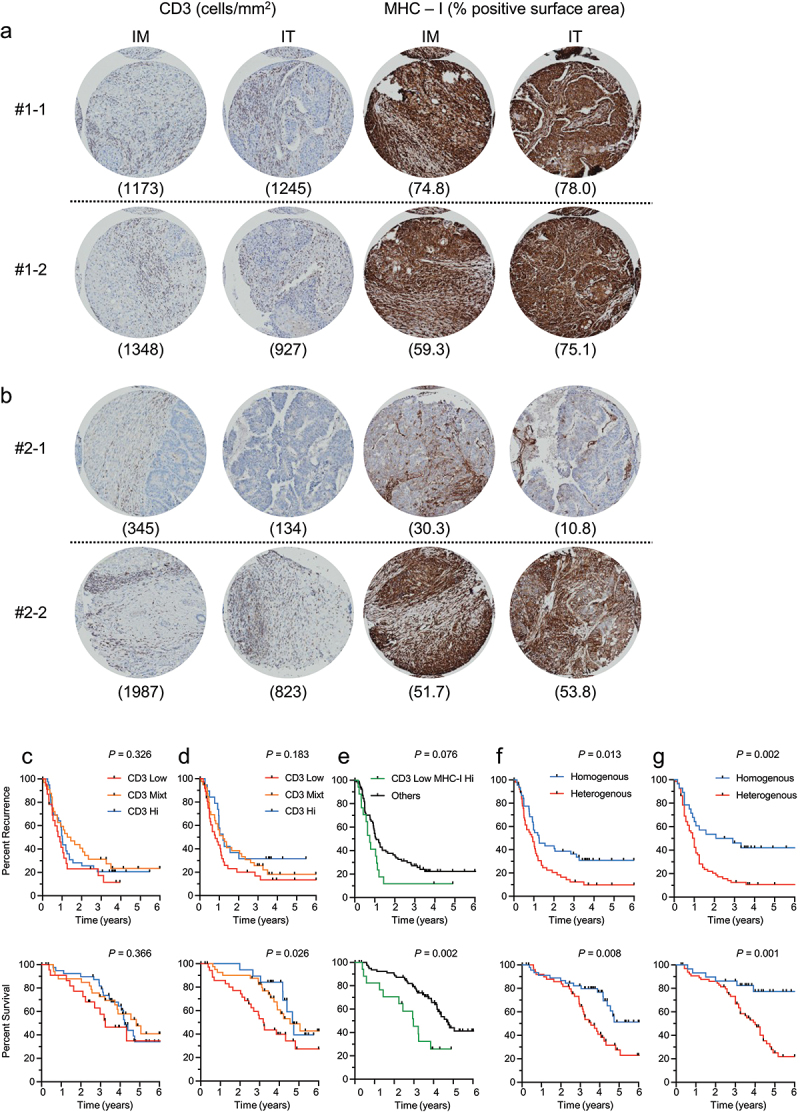
CD3^+^ T cells infiltration and MHC class I expression heterogeneity is associated with worse outcomes in patients with multiple colorectal cancer liver metastases (CRLM). Representative examples of two CRLMs from two patients. (a) in patient 1, the two CRLMs are homogeneously high (above cohort median) for CD3 and MHC-I at the infiltrative margin (IM) and intratumorally (IT). (b) in patient 2, the two CRLMs are heterogeneous for CD3 and MHC-I, one is low and the other is high in both the IM and IT. (Panel c-h) Kaplan–Meier curves are shown for time to recurrence (TTR) (top) and disease-specific survival (DSS) (bottom). (c) CD3 infiltration counted at the IM and IT and averaged across CRLM of a given patient; high patients are those with high CD3 in both IM and IT compartments, low patients are those with low CD3 in both compartments, and mixed patients are those with discordant high and low counts in these compartments, using the median of the cohort as cut-point. (d) CD3 infiltration is scored as in c, but each patient is classified according to the metastasis infiltrated by the lowest number of T cells, rather than the average. (e) presence in a given patient of at least one CRLM poorly infiltrated by CD3^+^ yet expressing high levels of MHC-I (CD3 low at the IM and IT, MHC-I high at the IM and IT). (f) patients grouped according to homogeneity or heterogeneity between CRLM for CD3^+^ T cell infiltration at the IM and IT, regardless of the degree of infiltration itself. (g) homogeneity or heterogeneity between CRLM for CD3^+^ T cell density and MHC-I expression at the IM and IT, regardless of the degree of CD3 infiltration or level of MHC-I expression itself. Log rank test.

Automated staining was performed on a VENTANA XT with the OmniMap DAB Detection System (ROCHE). High-resolution TMA digital images were acquired on Visiomorph v6 of Olympus BX61VS 20X scans, and quantification was carried out with Visiopharm Image Analysis Software (Molecular Devices) blinded to clinical data. The areas of positive signal and the total area of the tissue core were calculated on the basis of color, where pixels with identical RGB (red, green, and blue) values were grouped together to calculate a number of cells over total surface area for CD3 (Figure 1a) and a ratio of positive brown staining (moderate to strong) over total staining (all brown and hematoxylin blue) for MHC-I (Figure 1b). Mean ± standard error was calculated for each sixplicate at the IM and IT and the average was calculated per tumor.

### Statistical analysis

Disease-specific survival (DSS) and time to recurrence (TTR) were calculated from the time of hepatectomy using Kaplan–Meier method. Univariate Cox regression modeling was used to test the association between relevant clinicopathological and immune variables and patient outcome. CD3^+^ cell count and MHC-I percent surface expression were dichotomized in “high” and “low” categories using the median value from the entire reference cohort of 391 CRLMs, i.e.: 1016.7 cell/mm^2^ and 455.2 cell/mm^2^ for CD3 at the IM and IT, respectively, and 52.6% and 53.7% surface area for MHC-I at the IM and IT, respectively. Associations between patient outcome and CD3 or MHC-I were tested for each tumor area (IM and IT) and for patients grouped by scores of pathological response to chemotherapy. The relationship between patient outcome and homogeneity vs heterogeneity of a given immune parameter or pathological response score was also tested. Each patient was classified as bearing homogenous or heterogeneous metastases when their CRLMs fell in the same or different ‘’high’’ or ‘’low’’ immune or pathological response groups. A patient was globally classified as immune homogeneous only if the CD3 and the MHC-I in both IM and IT areas fell into the same category, either ‘’low’’ or ‘’high’’. A two-sided p-value of ≤ .05 was considered statistically significant (SPSS v. 24).

## Results

### Clinicopathological characteristics

We previously reported on the entire cohort of 214 patients with solitary and multiple metastases.^13,15^ Among these patients, 97 with multiple metastases were included in the current study. The median age at surgery was 64 years, and 69% were male. A median of three CRLMs of 2.6 cm were resected per patient. Neoadjuvant chemotherapy was administered to 87.6% of the patients most commonly as FOLFOX+ Bevacizumab for a median of six cycles. Most patients had moderate response to chemotherapy based on TRG (TRG 1–2 18.3%; TRG 3 28.0%; TRG 4–5 53.7%) and 5.2% had no residual cancer cell in their specimen as per the Blazer classification. The median follow-up time was 45 months. The median TTR was 12.4 months (95%CI 10.3–14.4), and the median DSS was 52.6 months (95%CI 46.1–59.1). Recurrence was seen in 73 (75.3%) patients (37.1% liver only, 14.4% lung only) and 49 (50.5%) patients had died of disease at the end of follow-up.

### Immune characteristics of CRLMs, homogeneity, and heterogeneity

Immune characteristics were analyzed in 220 CRLMs, 142 resected in 71 patients who were presented with 2–3 metastases, and 78 in 26 patients who presented with more than three metastases. The median CD3^+^ T cell density was 1044.1 cells/mm^2^ (range, 72.9–3373.3) in the IM area and 580.9 cells/mm^2^ (range, 32.4–4983.5) in the IT area. The median MHC-I surface area expression was 50.4% (range; 8.8–80.7) and 55.1% (range; 2.9–79.9) at the IM and IT areas, respectively. These median values were similar to those observed for the entire cohort that included 117 patients with single CRLMs (see methods).

Twenty-nine out of 96 (30.2%) patients bore CRLMs homogeneous for CD3 and MHC-I analyzed in both IM and IT areas (see Figure 1a–b for representative examples). Clinicopathological differences between immune homogenous and immune heterogeneous are presented at length in supplemental table S1. Immune heterogenous patients were more likely to have received pre-hepatectomy chemotherapy (92.5% vs. 75.9%, *p* = .013), presented more than three metastases (59.7% vs. 37.9%, *p* = .05), a positive resection margin (14.9% vs. 0.0%, *p* = .028), and an above median (5.2 ng/mL) pre-hepatectomy plasmatic CEA (53.4% vs. 28.6%, *p* = .05). Other clinicopathological characteristics were not significantly different between groups.

### Immune heterogeneity and outcomes

Different methods of classifying patients according to CD3^+^ T cell infiltration in CRLMs were compared for their ability to discriminate patients prognostically. We first averaged the CD3 counts of all CRLMs analyzed per patient and classified them according to the phenotypes as “low” (counts below median in both IT and IM areas), “high” (above median in both areas), and “mixt” (discordant high and low counts in these two areas). As shown in Figure 1c, averaging counts from multiple CRLMs could not discriminate patients by TTR or DSS. Since other publications^12^ supported that the metastasis least infiltrated by T cells could drive poorer outcomes, we next reclassified “low” patients to include 36 (37.5%) with at least one CRLM poorly infiltrated by CD3^+^ T cells both at the IM and IT areas (Figure 1d); by this method, shorter DSS was found in “low” patients compared to patients not bearing such a CRLM (38 vs. 56 months, *p* = .008), but did not reach significance for TTR. In the previous work,^13,16^ we had observed that CD3^+^ was prognostically discriminant as long as MHC-I was expressed in CRLMs and that particularly poor prognosis was observed in patients with low CD3^+^ T cells concurrent with high MHC-I expression. In this cohort, patients (*n* = 17) bearing at least one CRLM with the CD3^low^MHC-I^high^ phenotype compared to the rest of the cohort were found to have the shortest TTR (9 vs. 13 months, *p* = .075) and DSS (36 vs 56 months, *p* = .002) (Figure 1e).

We next compared the outcomes in patients according to the homogeneity or heterogeneity across multiple CRLMs classified as high or low for CD3^+^ T cells in both IM and IT areas. Patients (*n* = 45) with homogeneous CRLMs had longer TTR (15 vs 11 months, *p* = .013) and DSS (76 vs. 43 months, *p* = .008) compared to those with heterogeneous CRLMs (Figure 1f). When adding CD3 to the high or low expression of MHC-I in CRLMs in both IM and IT CRLMs as criteria for homogeneity, patients (*n* = 29) with homogeneous CRLMs had the longest TTR (30 vs 12 months, *p* = .002) and DSS (median not reached vs. 48 months, *p* = .001) compared to all other patients (Figure 1g).

To test whether the prognosis in immune homogeneous patients was not driven by an enriched population of highly infiltrated CRLM, we proceeded further to categorizing patients in ‘’low’’ vs. ‘’high’’ for CD3, based on the presence or absence of a poorly infiltrated CRLM. There were no significant differences in median TTR (35 vs. 19 months, *p* = .677) or DSS (both medians not reached, *p* = .851) in immune homogenous patients with or without a “low” CRLM, respectively. Likewise, for immune heterogeneous patients, there were no significant differences in median TTR (8 vs. 12 months, *p* = .297) or DSS (39 vs 51 months, *p* = .180) with or without a “low” CRLM, respectively (not shown). Overall, bearing metastases with similar immune features appeared to best predict good outcomes, while the poorly immunogenic features of a given metastasis tended to better identify patients with poor outcomes.

### Pathological response to preoperative chemotherapy and outcomes

**Figure 2.**
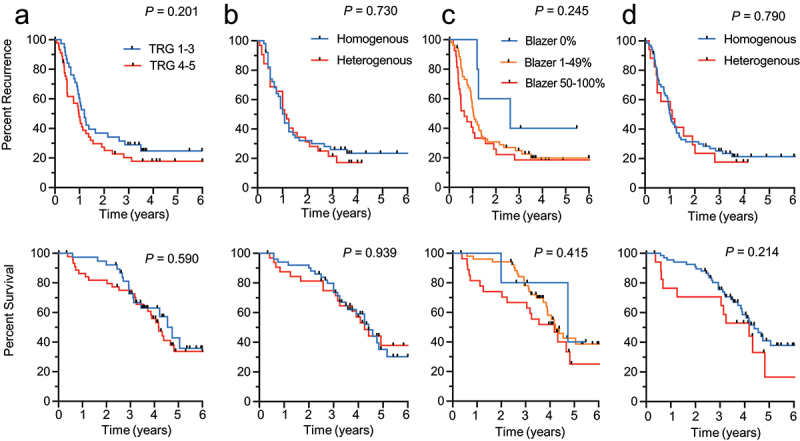
Outcomes according to homogeneity or heterogeneity in pathological response to pre-operative chemotherapy. Kaplan–Meier curves are shown for time to recurrence (TTR) (top) and disease-specific survival (DSS) (bottom). (a) Rubbia-Brandt tumor response grade (TRG) dichotomized in responders (TRG 1–3) and non-responders (TRG 4–5) according to their metastasis with the worst response. (b) patients grouped according to homogeneity or heterogeneity between metastases of the TRG score, using any difference in TRG score as the criteria to be classified as heterogeneous. (c) Blazer classification (0% vs. 1–49% vs. >50% residual cancer cells) using the metastasis with the worst response to classify patients. (d) patients grouped according to homogeneity or heterogeneity between metastases of the three categories of the Blazer score. Log rank test.

We evaluated the association between the TRG and Blazer scores, as well as their homogeneity, and outcomes (Figure 2). In contrast with the immune characteristics, a majority of patients had relative homogeneity in pathological response to chemotherapy. Sixty (61.9%) patients had the same TRG score and 69 (80.2%) had the same Blazer score across their CRLMs. Patients with the worst responses to preoperative chemotherapy by TRG and Blazer scores had relatively short median TTR (12 months and 8 months, respectively) and median DSS (both 50 months), but this was not statistically significant compared to patients with better scores (Figure 2a–c). Notably, heterogeneity in response to preoperative chemotherapy could not discriminate patients prognostically (Figure 2b–d). Consistently, TRG and Blazer scores were similarly distributed in patients grouped by immune homogeneous and heterogeneous CRLMs (Supplemental Table S1).

### Multivariate analysis

Table 1 summarizes the univariate survival analyses done for each clinicopathological and immune variable for TTR and DSS. A p-value of 0.10 was used as a cutoff to include variables in the multivariate model. The size of the largest metastasis ≥3 cm was associated with a shorter TTR (HR 1.486, *p* = .091) and shorter DSS (HR 1.704, *p* = .066). The high MHC-I expression was associated with shorter TTR (HR 1.774, *p* = .015 and HR 1.615, *p* = .043, in IM and IT, respectively) but was not significantly associated with DSS. There was no significant association between CD3 infiltration and either TTR or DSS. In univariate analysis, immune heterogeneity was associated with shorter TTR (HR 2.341, *p* = .003) and DSS (HR 3.859, *p* = .002). In multivariate analysis, only immune heterogeneity remained a significant predictor of shorter TTR (HR 2.612, *p* = .001) and DSS (HR 3.600, *p* = .004).

**Table 1.** Univariate and multivariate analysis of immune parameters and homogeneity and clinicopathological variables with outcome.

		Time to Recurrence (TTR)			Disease-Specific Survival (DSS)		
		HR	95% CI	P value	HR	95% CI	P value
**Univariate analysis**							
Age at surgery (≤65 vs >65 years)		1.114	[0.698–1.779]	.651	1.459	[0.827–2.573]	.193
Gender (male vs female)		0.799	[0.487–1.310]	.373	0.810	[0.449–1.459]	.482
Perioperative chemotherapy (none vs neoadjuvant)		1.211	[0.379–3.865]	.747	1.119	[0.271–4.623]	.876
Perioperative chemotherapy (none vs adjuvant)		1.346	[0.320–5.662]	.685	0.596	[0.084–4.241]	.605
Preoperative Avastin (no vs yes)		0.974	[0.481–1.972]	.941	0.817	[0.363–1.837]	.624
Rubbia-Brandt score (TRG 1–3 vs 4–5)		1.406	[0.854–2.313]	.180	1.226	[0.663–2.268]	.516
Blazer score (0–49% vs >50% residual cancer cells)		1.368	[0.851–2.201]	.196	1.446	[0.812–2.574]	.211
Number of metastases (<3 vs. ≥3)		1.245	[0.785–1.975]	.352	1.259	[0.713–2.223]	.427
Size of largest of metastases (<3 vs. ≥3 cm)		1.486	[0.938–2.355]	.091	1.704	[0.965–3.011]	.066
Margin of resection (negative vs positive)		1.207	[0.553–2.637]	.636	1.844	[0.826–4.117]	.135
Primary tumor site (colon vs rectum)		1.107	[0.691–1.771]	.673	1.296	[0.738–2.276]	.367
Primary tumor T stage (T1–2 vs. T3–4)		0.710	[0.352–1.431]	.338	0.814	[0.342–1.936]	.642
Primary tumor N stage (N0 vs N+)		1.441	[0.866–2.398]	.159	1.265	[0.667–2.398]	.472
CEA pre-hepatectomy (≤5.2 vs >5.2 ng/mL)		0.937	[0.568–1.545]	.798	0.933	[0.508–1.714]	.823
Fong Clinical Risk Score* (Low vs High CRS)		1.328	[0.817–2.159]	.252	1.224	[0.674–2.221]	.506
CD3 tumor infiltrating lymphocyte count							
	Interface (low vs high)	1.117	[.703–1.773]	.641	0.750	[.425–1.323]	.321
	Intratumoral (low vs high)	0.700	[.436–1.124]	.140	0.841	[.468–1.508]	.560
MHC-I expression surface area							
	Interface (low vs high)	1.774	[1.118–2.817]	.015	0.722	[.412–1.266]	.256
	Intratumoral (low vs high)	1.615	[1.015–2.570]	.043	1.179	[.668–2.079]	.570
Inter-metastatic immune profile of CD3 and MHC-I at the tumor interface and intratumorally (homogenous vs heterogenous)		2.341	[1.335–4.105]	.003	3.859	[1.639–9.089]	.002
**Multivariate analysis**							
Size of largest of metastases (<3 vs. ≥3 cm)		1.160	[0.73–1.86]	.530	1.330	[0.75–2.37]	.330
MHC-I expression surface area							
	Interface (low vs high)	1.610	[.96–2.68]	.069	-	-	-
	Intratumoral (low vs high)	1.450	[.87–2.44]	.160	-	-	-
Inter-metastatic immune profile of CD3 and MHC-I at the tumor interface and intratumorally (homogenous vs heterogenous)		2.612	[1.47–4.64]	.001	3.600	[1.51–8.58]	.004

Abbreviations: TRG, Tumor Regression Grade; T, Tumor; N, Node; CEA, Carcinoembryonic Antigen; MHC-I, Major Histocompatibility Complex class-I. *Clinical Risk Score calculated giving one point for each of the following clinicopathologic characteristics, and where low risk is defined as 0 to 2 points, high risk as 3 to 5 points: node-positive (N+) primary cancer, disease-free interval (time between resection of primary and liver recurrence) <12 months, more than one liver metastasis, largest liver metastasis >5 cm, and prehepatectomy serum carcinoembryonic antigen (CEA) level >200 ng/mL.

## Discussion

In this paper, we studied the prognostic implications of inter-tumoral immune heterogeneity in patients with multiple CRLMs resected with curative intent. Based on low vs. high CD3^+^ T cell infiltration and MHC-I expression at the infiltrative margin and within metastases, we found that immune homogeneity across CRLMs could best identify patients who recure less frequently and are likely to survive long term. We have confirmed previous findings in patients with multiple metastases that the least infiltrated CRLM, rather than the average count across metastases, is predictive of a poor prognosis.^12^ We also confirmed that high expression of MHC-I can identify poorly infiltrated CRLMs with worse prognoses, possibly due to immune escape mechanisms preventing T cell infiltration independently of the potential for antigen presentation by MHC-I molecules expressed by cancer cells.^13^ Combining CD3 and MHC-I to assess homogeneity was the best standalone prognostic marker and was superior to quantification based on CD3 T cell infiltrate alone. In contrast, homogeneity or heterogeneity in pathological response to pre-operative chemotherapy across metastases could not help distinguishing patients prognostically.

An ongoing challenge in the treatment of CRLM is to refine patient selection. Clinical prognostic models once greatly contributed to the understanding of this disease, but following modern advances in systemic and surgical treatments, they can no longer account for discrepancies seen between outcomes and clinicopathological characteristics. Discoveries regarding the molecular determinants of CRLM have brought precision to these tools. One example of prognostic score refinement is the addition of KRAS mutational status to the Clinical Risk Score, originally defined with standard clinical and pathological variables.^17^ Subsequent refinements, including additional relevant mutations, improved prognostic modeling but remain imperfect in part due to tumor heterogeneity in patients with multiple metastases.^18^

Our findings suggest that intra-patient, inter-metastatic immune heterogeneity bears important prognostic implications and that current pathological scores of responses to pre-operative chemotherapy may not well capture prognostically relevant heterogeneity. Tumor heterogeneity in metastatic colorectal cancer is implicated in mechanisms underlying resistance to systemic therapy and may be a deleterious consequence of somatic mutations accumulating in distinct metastases.^19,20^ However, mutational analyses between primary and metastatic CRC cannot completely account for overall cancer progression.^21^ The immune-selective evolutionary pressure and escape mechanisms of cancer cells in the tumor microenvironment represent an appealing hypothesis to better explain this process. Inter-metastatic immune heterogeneity may in fact be a surrogate marker for the development of aggressive forms of this cancer from immune privileged clones. A study of the immune landscape in metastatic CRC found recurrences bearing similar immunophenotypes to have a favorable prognosis, whereas changes in the immune infiltrate were associated with treatment failure.^22^

Genetic studies on intra-patient inter-metastatic heterogeneity have brought a better understanding to this phenomenon. Sveen *et al*.^2^ studied the number of DNA copy number aberrations in 123 CRLMs from 41 patients and showed that the 23 (56%) patients who had their metastatic deposits cluster in a patient-wise manner had significantly better outcomes (3-year OS 66% vs. 18%) than patients with high inter-metastatic genetic heterogeneity. They further investigated heterogeneity of the Consensus Molecular Subtype (CMS) in patients with multiple CRLMs and found that those with inter-metastatic CMS heterogeneity bore a higher number of lesions.^23^ The CMS heterogeneity itself as a variable had no significant prognostic value, but interestingly, neither did the CMS profile if selected randomly. Rather, classifying patients according to their “worst” CMS was the factor best able to stratify survival. An extreme example was found in a patient with 14 CRLMs with concordant CMS, but a single one with a poor prognostic profile. Interestingly, CMS has been associated with distinct immune phenotypes.^24^ This again speaks to the importance of analyzing every lesion in a given patient to truly understand the disease biology and predict its course.

The concept of tumor-infiltrating lymphocyte heterogeneity across multiple metastases and “worst” metastasis-driven prognosis in CRLM was well illustrated by Mlecnik *et al*.^*12*^ Our current study confirmed these findings and was able to show the added prognostic value of MHC-I when assessing immune heterogeneity. It is likely that this immune analytical scheme will be enhanced by the quantification of T cell subsets with distinct states of activation, cytotoxicity, proliferation, differentiation, stemness, exhaustion, tissue homing, and immune suppressive capacity (e.g. Tregs), combined with immune checkpoints expressed by cancer cells, macrophages, and other cells found in the tumor microenvironment. Mechanisms preventing T cells with adequate antigen-presentation capacity from infiltrating in metastases also deserve further work and may guide the development of novel therapeutic strategies.

A limitation of the current study resides in the potential for sampling bias related to TMA-type analyses. Previous studies have shown that in CRLMs, immune cell quantification using five to seven fields of 1 mm^2^ converges with whole-slide quantification.^25^ The use of 12 cores per CRLM and of a large sample number mitigates the risk that outliers skewed the study results. Whole slides were also analyzed to validate TMA-generated estimates (data not shown). An additional limitation is the lack of consistent mutational analysis for KRAS at the time of data collection, leading to missing data for analysis. In light of the growing knowledge regarding the prognostic value of genomic alterations, mutational status of all relevant genes should be included in future studies. Our findings require confirmation in larger independent cohorts.

## Conclusions

The present study supports the favorable prognosis of inter-metastatic immune homogeneity in patients bearing multiple CRLMs treated with curative intent. Analysis of T cell infiltration and MHC-I expression of all metastases in a given patient appears necessary as a single metastasis with poor immune features out of several other metastases with better immune features is still predictive of a poor outcome.

## Supplementary Material

Supplemental Material

## Data Availability

All raw tissue microarray images and associated marker quantification and patient clinicopathological characteristics are available upon request from the study authors.
